# 1,1,2,2,2,3,3,3-Octa­carbonyl-1,1,2,3-tetra­kis­(1,3,5-tri­aza-7-phosphatri­cyclo­[3.3.1.1^3,7^]decane-κ*P*)-*triangulo*-triosmium(0)

**DOI:** 10.1107/S2414314620009359

**Published:** 2020-07-17

**Authors:** David M. Marolf, Kristen L. Brehm, Vincent M. Lynch, Gregory L. Powell

**Affiliations:** a Department of Chemistry & Biochemistry, Abilene Christian University, Abilene, Texas 79699-8132, USA; b Department of Chemistry, University of Texas, Austin, Texas 78712, USA; Vienna University of Technology, Austria

**Keywords:** crystal structure, osmium carbon­yl, trinuclear cluster, PTA ligands

## Abstract

The title compound, [Os_3_(C_6_H_12_N_3_P)_4_(CO)_8_], crystallizes in the ortho­rhom­bic space group *Pbca* with *Z* = 8. The mol­ecule consists of a triangular triosmium(0) core surrounded by eight carbonyl ligands and four 1,3,5-tri­aza-7-phosphatri­cyclo­[3.3.1.1^3,7^]decane (or PTA) ligands. One Os atom is coordinated by two PTA ligands and two CO ligands, while the other two Os atoms are each bonded to a single PTA ligand and three CO ligands. There is a small disorder associated with the Os_3_ unit so that a minor orientation has an occupancy of 2.17 (4)%. The title compound represents the first structurally characterized triangular Os_3_ carbonyl cluster with four monodentate tertiary phosphane ligands.

## Abstract 2

The title mol­ecule consists of a triangular triosmium(0) core surrounded by eight carbonyl ligands and four 1,3,5-tri­aza-7-phosphatri­cyclo­[3.3.1.1^3,7^]decane ligands.

## Graphical abstract

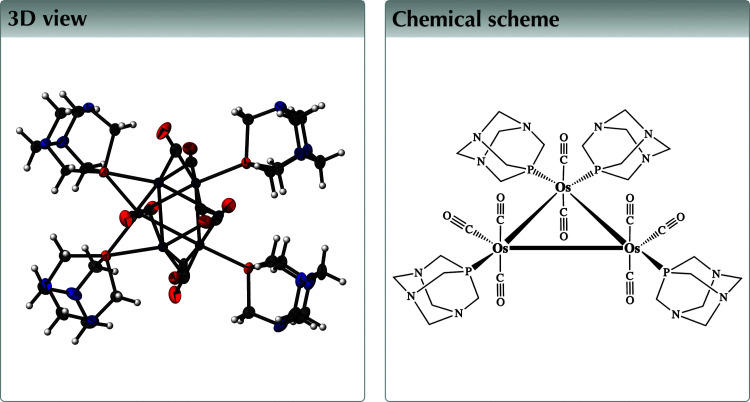

## Structure description

The water-soluble, air-stable, and non-toxic phosphane ligand 1,3,5-tri­aza-7-phosphatri­cyclo­[3.3.1.1^3,7^]decane (PTA) is often used in attempts to prepare metal complexes that are soluble in water (Phillips *et al.*, 2004 ▸; Bravo *et al.*, 2010 ▸). A series of triangular trinuclear metal carbonyl cluster complexes with the formula *M*_3_(CO)_12–*x*_(PTA)_*x*_, where *M* = Ru or Os and *x* = 1, 2, or 3, was recently synthesized by reactions of PTA with *M*_3_(CO)_12_ (Mager *et al.*, 2015 ▸; Dugan *et al.*, 2016 ▸; Naza­rov *et al.*, 2016 ▸). The complexes *M*_3_(CO)_11_(PTA) and *M*_3_(CO)_10_(PTA)_2_ are insoluble in water, while the complexes *M*_3_(CO)_9_(PTA)_3_ dissolve in water with a pH lower than 4. The title complex, Os_3_(CO)_8_(PTA)_4_, was also prepared and found to be soluble under acidic, neutral and basic aqueous conditions (Dugan *et al.*, 2016 ▸). As a result, tests are currently underway to determine if the title complex displays anti­cancer activity. An X-ray crystallographic analysis of Os_3_(CO)_8_(PTA)_4_ is warranted because there are no reports describing the crystal structure of a triosmium carbonyl cluster containing four monodentate phosphane ligands. Previous efforts to produce triangular triosmium carbonyl compounds with more than three phosphane ligands have typically resulted in cluster fragmentation (Alex *et al.*, 1987 ▸).

**Figure 1:**
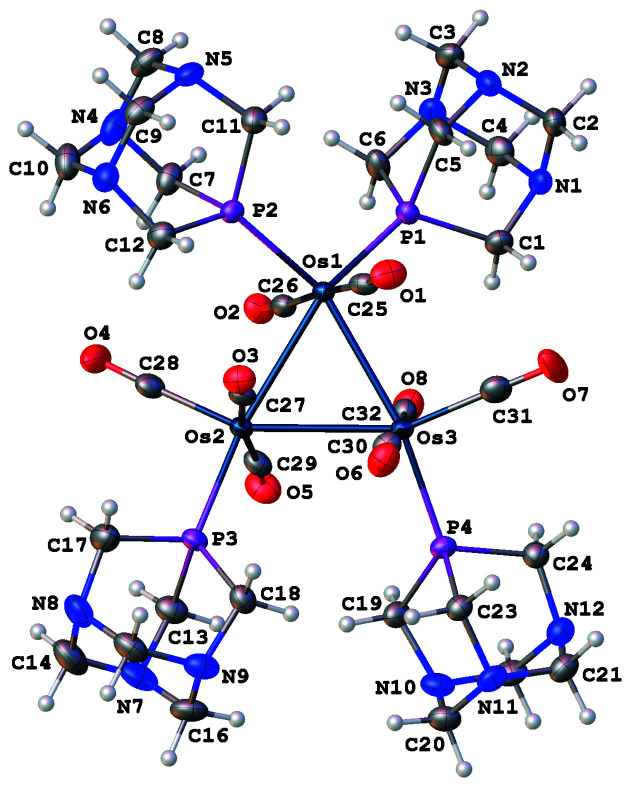
View of the title mol­ecule showing the atom-labeling scheme. Displacement ellipsoids are scaled to the 50% probability level. The Os_3_ core is represented by the major disorder component. For the sake of clarity, the atoms labels for C15 (in front of N7) and C22 (behind N11) are omitted.

In the title compound, the four phosphane ligands adopt positions that maximize the distance between them (Fig. 1 ▸). Two PTA ligands coordinate to Os1 through their P atoms, and the other two PTA ligands coordinate one each to Os2 and Os3 through their P atoms. All PTA ligands are located in equatorial coordination sites so that the four phospho­rus atoms are within 1 Å of residing in the same plane as the three osmium atoms. Two carbonyl ligands occupy equatorial sites (one each on Os2 and Os3), while the other six CO ligands occupy axial sites (two per Os atom). Each Os atom exhibits a pseudo-octa­hedral coordination environment, but the three coordination spheres are twisted relative to one another so that the axial CO ligands are no longer perpendicular to the Os3 plane as they are in Os_3_(CO)_12_ (Corey & Dahl, 1962 ▸). The average C_ax_—Os—Os—C_ax_ torsion angle is 29 (3)°. Crystal structures have been reported for five Ru_3_(CO)_12–_ *_x_L_x_* complexes: one [*L* = P(OMe)_2_Ph] with two bridging CO ligands (Bruce *et al.*, 1985 ▸), two [*L* = P(OEt)_3_, PMe_2_Ph] with two semi-bridging CO ligands, and two [*L* = P(OMe)_3_, P(OPh)_3_] with only terminal CO ligands (Bruce *et al.*, 1989 ▸). In all cases, the phosphane or phosphite ligands adopt the same coordination geometry as in the title complex. The average C_ax_—Ru—Ru—C_ax_ torsion angles in the latter two are similar to that in the title complex with 35 (3)° for *L* = P(OMe)_3_ and 30 (2)° for *L* = P(OPh)_3_. Such torsional twisting was also noted in the cases of Ru_3_(CO)_9_(PTA)_3_ and Os_3_(CO)_9_(PTA)_3_, albeit to a significantly lower degree with average C_ax_—*M*—*M*—C_ax_ torsion angles of 19 (2) and 17 (2)°, respectively (Mager *et al.*, 2015 ▸; Dugan *et al.*, 2016 ▸). The average Os—Os bond length of 2.903 (22) Å in the title complex is virtually the same as that of 2.90 (2) Å in Os_3_(CO)_9_(PTA)_3_, suggesting that torsional distortion is preferred over metal–metal bond lengthening.

**Figure 2:**
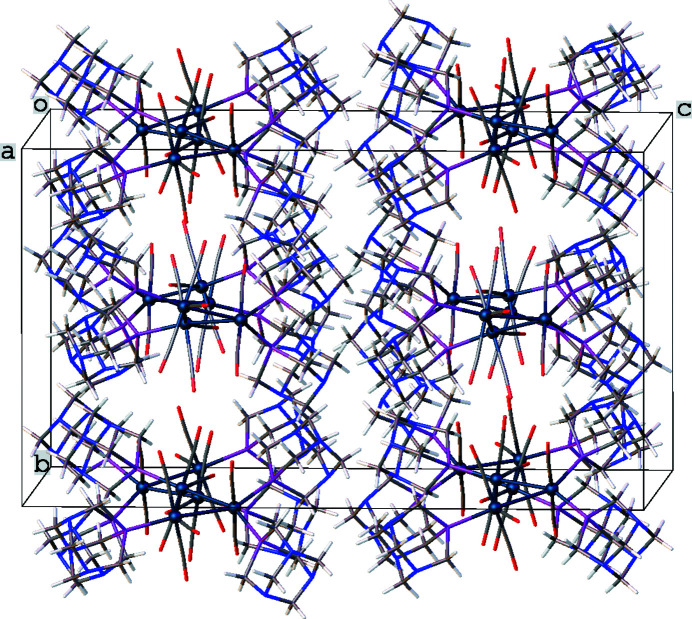
Packing of the mol­ecules viewed approximately along the *a* axis.

**Figure 3:**
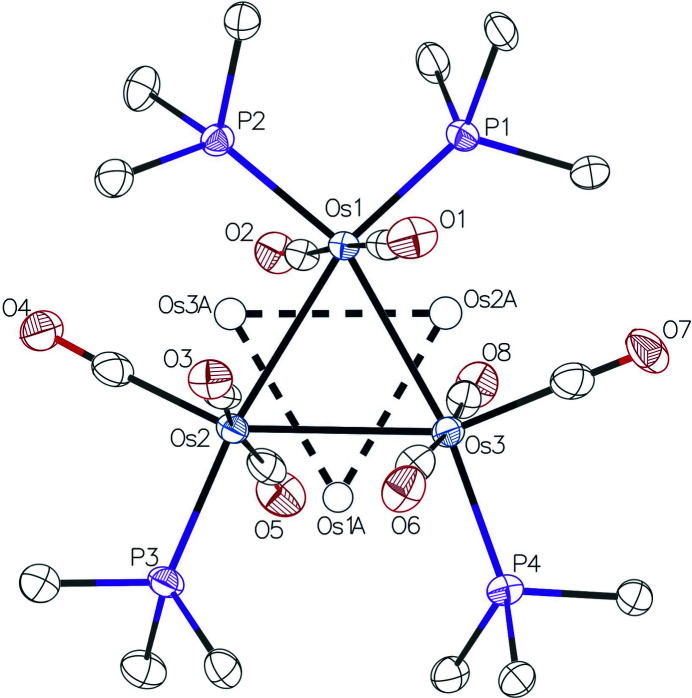
View of the major and minor components of the disordered triangular Os_3_ unit.

In the structure of the title complex, the mol­ecules are stacked parallel to the *a* axis of the unit cell with every other Os_3_ triangle facing in the opposite direction and tilted at an angle of 9 (1)° from the previous Os_3_ triangle in the same stack (Fig. 2 ▸). The planes defined by the Os_3_ units appear to be roughly perpendicular to the *b* axis, but actually form 21 (1)° angles with the *ac* plane. There is a slight disorder in the title complex associated with the triangular Os_3_ unit so that two different orientations are observed with a 0.9783 (4)/0.0217 (4) ratio of major-to-minor components (Fig. 3 ▸). This type of disorder is rather common in *M*_3_(CO)_12–_ *_x_L_x_* complexes in which *M* = Ru or Os. For example, a 50/50 disorder of the *M*_3_ unit exists in Os_3_(CO)_6_[P(OMe)_3_]_6_, Ru_3_(CO)_8_(PMe_2_Ph)_4_, and Ru_3_(CO)_8_[P(OEt)_3_]_4_, while an 85/15 disorder is present in Ru_3_(CO)_8_[P(OMe)_3_]_4_ (Alex *et al.*, 1987 ▸; Bruce *et al.*, 1989 ▸).

## Synthesis and crystallization

Dodceca­carbonyl­triosmium (63.3 mg, 0.0698 mmol), PTA (87.6 mg, 0.557 mmol), 1,2-di­chloro­benzene (6 ml), and aceto­nitrile (2 ml) were added to a 35 ml glass reaction vessel, then sealed with a PTFE cap and placed in a CEM Discover-SP microwave reactor. The mixture was stirred and heated at 458 K for 24 min to produce a vibrant orange solution. The solvent was removed and water (15 ml) was added to dissolve the residue. The resulting solution was filtered through a glass frit and the orange filtrate collected. After 16 h, a precipitate had formed. Filtering again allowed for the isolation of the title complex as an orange solid. IR (νCO cm^−1^ in CHCl_3_): 2033(*w*), 1970(*sh*), 1953(*vs*), 1921(*m*). Crystals grew as thin, reddish orange plates *via* diffusion of *n*-hexane into a CH_2_Cl_2_ solution.

## Refinement

Crystal data, data collection and structure refinement details are summarized in Table 1 ▸. Three rather large electron density peaks located near the Os atoms persisted in the difference electron-density map after all of the expected atoms were included in the model. The position of these peaks suggested there was a slight disorder in the complex resulting from an approximately 60° rotation about an axis perpendicular to the plane through the three Os atoms. A disorder model with this in mind was proposed where these three electron-density peaks represented the alternate orientation of the Os_3_ core. The variable *x* was assigned to the site occupancy for Os1, Os2 and Os3, while the site occupancy for Os1*A*, Os2*A* and Os3*A* was set to (1 − *x*). The displacement parameters for the lower occupancy Os atoms were set to be equal to those of the major component. The variable *x* refined to 0.9783 (4). Disorder of the associated CO and PTA ligands could not be resolved.

## Supplementary Material

Crystal structure: contains datablock(s) I. DOI: 10.1107/S2414314620009359/wm4135sup1.cif

Structure factors: contains datablock(s) I. DOI: 10.1107/S2414314620009359/wm4135Isup2.hkl

Supporting information file. DOI: 10.1107/S2414314620009359/wm4135Isup4.cml

CCDC reference: 1949128

Additional supporting information:  crystallographic information; 3D view; checkCIF report

## Figures and Tables

**Table 1:** Experimental details

Crystal data	
Chemical formula	[Os_3_(C_6_H_12_N_3_P)_4_(CO)_8_]
*M*_r_	1423.30
Crystal system, space group	Orthorhombic, *P* *b* *c* *a*
Temperature (K)	100
*a*, *b*, *c* (Å)	18.7500 (2), 15.8889 (2), 27.5984 (3)
*V* (Å^3^)	8222.03 (16)
*Z*	8
Radiation type	Cu *K*α
μ (mm^−1^)	19.16
Crystal size (mm)	0.18 × 0.09 × 0.01

Data collection	
Diffractometer	Agilent SuperNova with AtlasS2 CCD
Absorption correction	Gaussian (*CrysAlis PRO*; Agilent, 2016 ▸)
*T*_min_, *T*_max_	0.142, 0.783
No. of measured, independent and observed [*I* > 2σ(*I*)] reflections	38755, 8184, 7535
*R*_int_	0.048
(sin θ/λ)_max_ (Å^−1^)	0.624

Refinement	
*R*[*F* ^2^ > 2σ(*F* ^2^)], *wR*(*F* ^2^), *S*	0.032, 0.076, 1.14
No. of reflections	8184
No. of parameters	542
H-atom treatment	H-atom parameters constrained
Δρ_max_, Δρ_min_ (e Å^−3^)	1.24, −1.34

Computer programs: *CrysAlis PRO* (Agilent, 2016 ▸), *SUPERFLIP* (Palatinus & Chapuis, 2007 ▸), *SHELXL2014/7* (Sheldrick 2015 ▸) and *XP* in *SHELXTL/PC* (Sheldrick, 2008 ▸).

## full crystallographic data

### Crystal data

**Table:**

[Os_3_(C_6_H_12_N_3_P)_4_(CO)_8_]	*D*_x_ = 2.300 Mg m^−^^3^
*M**_r_* = 1423.30	Cu *K*α radiation, λ = 1.54184 Å
Orthorhombic, *P**b**c**a*	Cell parameters from 21014 reflections
*a* = 18.7500 (2) Å	θ = 3.9–74.1°
*b* = 15.8889 (2) Å	µ = 19.16 mm^−^^1^
*c* = 27.5984 (3) Å	*T* = 100 K
*V* = 8222.03 (16) Å^3^	Plates, red-orange
*Z* = 8	0.18 × 0.09 × 0.01 mm
*F*(000) = 5408	

### Data collection

**Table:**

Agilent SuperNova with AtlasS2 CCD diffractometer	7535 reflections with *I* > 2σ(*I*)
Radiation source: sealed microfocus tube	*R*_int_ = 0.048
ω–scans	θ_max_ = 74.3°, θ_min_ = 3.2°
Absorption correction: gaussian (CrysAlisPro; Agilent, 2016)	*h* = −23→23
*T*_min_ = 0.142, *T*_max_ = 0.783	*k* = −19→19
38755 measured reflections	*l* = −33→33
8184 independent reflections	

### Refinement

**Table:**

Refinement on *F*^2^	0 restraints
Least-squares matrix: full	Hydrogen site location: inferred from neighbouring sites
*R*[*F*^2^ > 2σ(*F*^2^)] = 0.032	H-atom parameters constrained
*wR*(*F*^2^) = 0.076	*w* = 1/[σ^2^(*F*_o_^2^) + (0.0225*P*)^2^ + 60.4468*P*] where *P* = (*F*_o_^2^ + 2*F*_c_^2^)/3
*S* = 1.14	(Δ/σ)_max_ = 0.002
8184 reflections	Δρ_max_ = 1.24 e Å^−^^3^
542 parameters	Δρ_min_ = −1.34 e Å^−^^3^

### Special details

**Table:**

Geometry. All esds (except the esd in the dihedral angle between two l.s. planes) are estimated using the full covariance matrix. The cell esds are taken into account individually in the estimation of esds in distances, angles and torsion angles; correlations between esds in cell parameters are only used when they are defined by crystal symmetry. An approximate (isotropic) treatment of cell esds is used for estimating esds involving l.s. planes.
Refinement. Three rather large peaks persisted in the difference electron density map after all the expected atoms were included in the model. These peaks, all greater than 4 e-/A^3^, were located near the Os atoms. The position of these peaks suggested there was a slight disorder in the complex. The disorder resulted from an approximately 60 degree rotation about an axis perpendicular to the plane through the three Os atoms. A disorder model with this in mind was proposed where these three peaks represented the alternate orientation of the Os atoms. The variable x was assigned to the site occupancy for Os1, Os2 and Os3, while the site occupancy factors for Os1a, Os2a and Os3a was set to (1-x). The displacement parameters for the lower occupancy Os atoms was set to be equal to that of the major component. The variable x refined to 0.9783 (4). The other atoms of the alternate component were not included in the disorder model because the very low electron density of a 2% component would be lost in the noise.

### Fractional atomic coordinates and isotropic or equivalent isotropic displacement parameters (Å^2^)

**Table:**

	*x*	*y*	*z*	*U*_iso_*/*U*_eq_	Occ. (<1)
Os1	0.13660 (2)	0.51582 (2)	0.16473 (2)	0.01732 (6)	0.9783 (4)
Os1A	0.1385 (5)	0.5007 (6)	0.2876 (4)	0.017*	0.0217 (4)
Os2	0.06461 (2)	0.53429 (2)	0.25694 (2)	0.01999 (7)	0.9783 (4)
Os2A	0.2036 (6)	0.4728 (7)	0.1943 (4)	0.020*	0.0217 (4)
Os3	0.21112 (2)	0.47110 (2)	0.25254 (2)	0.01941 (7)	0.9783 (4)
Os3A	0.0633 (6)	0.5501 (7)	0.2014 (4)	0.019*	0.0217 (4)
P1	0.19602 (7)	0.44377 (9)	0.10564 (5)	0.0205 (3)	
P2	0.06469 (7)	0.59975 (9)	0.11836 (5)	0.0204 (3)	
P3	0.03369 (8)	0.56927 (10)	0.33489 (5)	0.0231 (3)	
P4	0.24364 (8)	0.41978 (9)	0.32711 (5)	0.0215 (3)	
O1	0.2497 (2)	0.6564 (3)	0.16584 (15)	0.0343 (10)	
O2	0.0236 (2)	0.3749 (3)	0.16316 (15)	0.0288 (9)	
O3	0.1199 (2)	0.7154 (3)	0.24106 (15)	0.0298 (9)	
O4	−0.0818 (2)	0.5625 (4)	0.21312 (16)	0.0422 (12)	
O5	0.0292 (3)	0.3491 (3)	0.28011 (17)	0.0379 (11)	
O6	0.2521 (2)	0.6475 (3)	0.28886 (16)	0.0354 (10)	
O7	0.3580 (2)	0.4501 (4)	0.20884 (17)	0.0442 (12)	
O8	0.1579 (2)	0.2948 (3)	0.22133 (16)	0.0336 (10)	
N1	0.2921 (3)	0.3214 (3)	0.07410 (17)	0.0263 (10)	
N2	0.2723 (3)	0.4408 (3)	0.01888 (18)	0.0291 (11)	
N3	0.1794 (3)	0.3322 (3)	0.02862 (18)	0.0310 (11)	
N4	−0.0582 (3)	0.6146 (3)	0.0632 (2)	0.0350 (12)	
N5	0.0450 (3)	0.6985 (3)	0.03545 (17)	0.0287 (11)	
N6	−0.0219 (3)	0.7418 (3)	0.10798 (19)	0.0297 (11)	
N7	−0.0301 (3)	0.5304 (4)	0.4224 (2)	0.0414 (14)	
N8	−0.0477 (3)	0.6759 (4)	0.3919 (2)	0.0389 (13)	
N9	0.0689 (3)	0.6327 (3)	0.42524 (18)	0.0327 (12)	
N10	0.2154 (3)	0.3296 (3)	0.41086 (17)	0.0302 (11)	
N11	0.3084 (3)	0.4396 (3)	0.41678 (17)	0.0279 (11)	
N12	0.3330 (3)	0.3066 (3)	0.37345 (18)	0.0269 (10)	
C1	0.2665 (3)	0.3640 (4)	0.1184 (2)	0.0276 (12)	
H1A	0.3073	0.3920	0.1345	0.033*	
H1B	0.2473	0.3213	0.1410	0.033*	
C2	0.3233 (3)	0.3817 (4)	0.0398 (2)	0.0308 (13)	
H2A	0.3611	0.4140	0.0566	0.037*	
H2B	0.3463	0.3500	0.0131	0.037*	
C3	0.2143 (3)	0.3926 (4)	−0.0037 (2)	0.0342 (14)	
H3A	0.1780	0.4326	−0.0158	0.041*	
H3B	0.2337	0.3619	−0.0319	0.041*	
C4	0.2333 (3)	0.2771 (4)	0.0496 (2)	0.0307 (13)	
H4A	0.2536	0.2417	0.0235	0.037*	
H4B	0.2100	0.2392	0.0732	0.037*	
C5	0.2442 (3)	0.4983 (4)	0.0563 (2)	0.0282 (13)	
H5A	0.2115	0.5391	0.0407	0.034*	
H5B	0.2844	0.5305	0.0704	0.034*	
C6	0.1397 (3)	0.3753 (4)	0.0674 (2)	0.0300 (13)	
H6A	0.1166	0.3325	0.0882	0.036*	
H6B	0.1015	0.4099	0.0525	0.036*	
C7	−0.0174 (3)	0.5548 (4)	0.0924 (2)	0.0293 (13)	
H7A	−0.0046	0.5059	0.0720	0.035*	
H7B	−0.0480	0.5341	0.1191	0.035*	
C8	−0.0167 (4)	0.6460 (4)	0.0227 (2)	0.0346 (14)	
H8A	−0.0486	0.6793	0.0015	0.042*	
H8B	0.0004	0.5973	0.0036	0.042*	
C9	0.0183 (3)	0.7678 (4)	0.0658 (2)	0.0282 (13)	
H9A	0.0594	0.8020	0.0767	0.034*	
H9B	−0.0124	0.8044	0.0456	0.034*	
C10	−0.0806 (3)	0.6871 (4)	0.0926 (3)	0.0369 (15)	
H10A	−0.1150	0.7210	0.0736	0.044*	
H10B	−0.1057	0.6661	0.1217	0.044*	
C11	0.0986 (3)	0.6478 (4)	0.0616 (2)	0.0285 (13)	
H11A	0.1398	0.6842	0.0696	0.034*	
H11B	0.1157	0.6025	0.0400	0.034*	
C12	0.0250 (3)	0.6981 (4)	0.1431 (2)	0.0295 (13)	
H12A	−0.0029	0.6843	0.1725	0.035*	
H12B	0.0639	0.7367	0.1528	0.035*	
C13	−0.0117 (4)	0.4926 (4)	0.3748 (2)	0.0326 (14)	
H13A	−0.0558	0.4726	0.3587	0.039*	
H13B	0.0198	0.4434	0.3799	0.039*	
C14	−0.0783 (4)	0.6017 (5)	0.4161 (3)	0.0469 (19)	
H14A	−0.0957	0.6191	0.4485	0.056*	
H14B	−0.1201	0.5825	0.3972	0.056*	
C15	0.0165 (4)	0.7002 (4)	0.4186 (2)	0.0368 (15)	
H15A	0.0021	0.7213	0.4509	0.044*	
H15B	0.0398	0.7473	0.4012	0.044*	
C16	0.0344 (4)	0.5607 (5)	0.4480 (2)	0.0382 (16)	
H16A	0.0211	0.5762	0.4815	0.046*	
H16B	0.0692	0.5140	0.4499	0.046*	
C17	−0.0309 (4)	0.6568 (4)	0.3413 (2)	0.0351 (14)	
H17A	−0.0109	0.7077	0.3257	0.042*	
H17B	−0.0754	0.6418	0.3240	0.042*	
C18	0.1000 (3)	0.6076 (4)	0.3782 (2)	0.0283 (13)	
H18A	0.1358	0.5628	0.3839	0.034*	
H18B	0.1251	0.6565	0.3640	0.034*	
C19	0.1803 (3)	0.3634 (4)	0.3669 (2)	0.0269 (12)	
H19A	0.1417	0.4024	0.3767	0.032*	
H19B	0.1584	0.3164	0.3486	0.032*	
C20	0.2474 (4)	0.3985 (4)	0.4397 (2)	0.0311 (13)	
H20A	0.2102	0.4413	0.4461	0.037*	
H20B	0.2627	0.3754	0.4714	0.037*	
C21	0.3610 (3)	0.3745 (4)	0.4040 (2)	0.0296 (13)	
H21A	0.4014	0.4016	0.3869	0.036*	
H21B	0.3800	0.3496	0.4342	0.036*	
C22	0.2710 (3)	0.2698 (4)	0.3978 (2)	0.0300 (13)	
H22A	0.2501	0.2265	0.3763	0.036*	
H22B	0.2875	0.2412	0.4276	0.036*	
C23	0.2860 (3)	0.4874 (4)	0.3736 (2)	0.0239 (12)	
H23A	0.3281	0.5154	0.3593	0.029*	
H23B	0.2518	0.5316	0.3835	0.029*	
C24	0.3136 (3)	0.3386 (4)	0.3252 (2)	0.0255 (12)	
H24A	0.2969	0.2911	0.3049	0.031*	
H24B	0.3567	0.3625	0.3096	0.031*	
C25	0.2074 (3)	0.6038 (4)	0.16749 (19)	0.0253 (12)	
C26	0.0661 (3)	0.4266 (4)	0.1660 (2)	0.0253 (12)	
C27	0.1014 (3)	0.6473 (4)	0.24557 (19)	0.0252 (12)	
C28	−0.0263 (3)	0.5543 (4)	0.2306 (2)	0.0295 (13)	
C29	0.0443 (3)	0.4157 (4)	0.2700 (2)	0.0279 (13)	
C30	0.2336 (3)	0.5838 (4)	0.2740 (2)	0.0258 (12)	
C31	0.3020 (3)	0.4568 (4)	0.2250 (2)	0.0315 (14)	
C32	0.1761 (3)	0.3613 (4)	0.2316 (2)	0.0273 (12)	

### Atomic displacement parameters (Å^2^)

**Table:**

	*U*^11^	*U*^22^	*U*^33^	*U*^12^	*U*^13^	*U*^23^
Os1	0.01825 (11)	0.01878 (12)	0.01494 (12)	0.00020 (9)	0.00030 (8)	−0.00033 (8)
Os2	0.01924 (12)	0.02431 (13)	0.01642 (12)	−0.00041 (10)	0.00185 (9)	−0.00012 (9)
Os3	0.01938 (12)	0.02142 (13)	0.01743 (12)	−0.00041 (9)	−0.00109 (8)	0.00100 (9)
P1	0.0214 (6)	0.0223 (7)	0.0178 (6)	0.0011 (6)	0.0014 (5)	−0.0025 (5)
P2	0.0193 (6)	0.0222 (7)	0.0198 (6)	−0.0015 (5)	−0.0012 (5)	0.0016 (5)
P3	0.0234 (7)	0.0283 (7)	0.0175 (6)	−0.0014 (6)	0.0037 (5)	−0.0003 (6)
P4	0.0235 (7)	0.0227 (7)	0.0183 (6)	−0.0024 (6)	−0.0023 (5)	0.0011 (5)
O1	0.035 (2)	0.034 (2)	0.034 (2)	−0.018 (2)	−0.0030 (19)	0.0064 (19)
O2	0.025 (2)	0.029 (2)	0.032 (2)	−0.0116 (18)	0.0010 (17)	−0.0036 (17)
O3	0.037 (2)	0.023 (2)	0.029 (2)	−0.0034 (19)	−0.0014 (18)	−0.0023 (17)
O4	0.022 (2)	0.072 (3)	0.033 (2)	−0.008 (2)	−0.0003 (18)	0.014 (2)
O5	0.044 (3)	0.028 (2)	0.041 (3)	−0.008 (2)	0.010 (2)	0.004 (2)
O6	0.036 (2)	0.029 (2)	0.041 (3)	−0.001 (2)	−0.010 (2)	−0.005 (2)
O7	0.022 (2)	0.076 (4)	0.034 (3)	−0.008 (2)	0.0049 (19)	−0.020 (2)
O8	0.035 (2)	0.026 (2)	0.040 (2)	−0.0007 (19)	−0.0056 (19)	−0.0040 (19)
N1	0.026 (2)	0.030 (3)	0.023 (2)	0.009 (2)	0.0000 (19)	−0.002 (2)
N2	0.028 (3)	0.034 (3)	0.025 (2)	0.008 (2)	0.005 (2)	0.002 (2)
N3	0.028 (3)	0.038 (3)	0.026 (3)	0.004 (2)	−0.003 (2)	−0.012 (2)
N4	0.023 (2)	0.032 (3)	0.050 (3)	−0.002 (2)	−0.011 (2)	0.006 (3)
N5	0.030 (3)	0.035 (3)	0.022 (2)	0.001 (2)	−0.005 (2)	0.006 (2)
N6	0.028 (3)	0.028 (3)	0.032 (3)	0.005 (2)	−0.001 (2)	0.002 (2)
N7	0.045 (3)	0.049 (4)	0.030 (3)	−0.014 (3)	0.016 (3)	−0.002 (3)
N8	0.034 (3)	0.046 (3)	0.036 (3)	0.006 (3)	0.010 (2)	−0.008 (3)
N9	0.040 (3)	0.035 (3)	0.023 (3)	−0.010 (2)	0.002 (2)	−0.002 (2)
N10	0.038 (3)	0.032 (3)	0.021 (2)	−0.007 (2)	0.003 (2)	0.003 (2)
N11	0.029 (3)	0.030 (3)	0.025 (3)	−0.003 (2)	−0.010 (2)	0.000 (2)
N12	0.027 (2)	0.026 (2)	0.029 (3)	−0.001 (2)	−0.003 (2)	0.002 (2)
C1	0.029 (3)	0.033 (3)	0.021 (3)	0.001 (3)	−0.004 (2)	−0.003 (2)
C2	0.022 (3)	0.042 (4)	0.029 (3)	0.008 (3)	0.002 (2)	−0.001 (3)
C3	0.036 (3)	0.044 (4)	0.022 (3)	0.011 (3)	−0.002 (2)	−0.005 (3)
C4	0.035 (3)	0.026 (3)	0.031 (3)	0.004 (3)	0.002 (3)	−0.004 (3)
C5	0.025 (3)	0.032 (3)	0.028 (3)	0.005 (3)	0.009 (2)	0.000 (2)
C6	0.022 (3)	0.037 (3)	0.031 (3)	0.003 (3)	0.001 (2)	−0.012 (3)
C7	0.021 (3)	0.026 (3)	0.040 (3)	−0.003 (2)	−0.004 (2)	0.005 (3)
C8	0.039 (4)	0.035 (3)	0.030 (3)	0.004 (3)	−0.015 (3)	−0.002 (3)
C9	0.029 (3)	0.024 (3)	0.032 (3)	−0.005 (2)	−0.008 (2)	0.006 (2)
C10	0.023 (3)	0.036 (3)	0.052 (4)	0.003 (3)	0.004 (3)	0.008 (3)
C11	0.025 (3)	0.032 (3)	0.028 (3)	0.002 (3)	0.001 (2)	0.004 (3)
C12	0.032 (3)	0.028 (3)	0.028 (3)	0.004 (3)	−0.002 (2)	−0.001 (2)
C13	0.034 (3)	0.035 (3)	0.028 (3)	−0.008 (3)	0.005 (3)	0.000 (3)
C14	0.036 (4)	0.064 (5)	0.041 (4)	−0.007 (4)	0.015 (3)	−0.012 (4)
C15	0.041 (4)	0.039 (4)	0.030 (3)	−0.004 (3)	0.012 (3)	−0.007 (3)
C16	0.050 (4)	0.047 (4)	0.018 (3)	−0.004 (3)	0.001 (3)	0.000 (3)
C17	0.037 (3)	0.040 (4)	0.028 (3)	0.008 (3)	0.000 (3)	−0.002 (3)
C18	0.024 (3)	0.031 (3)	0.029 (3)	−0.002 (3)	0.004 (2)	−0.003 (2)
C19	0.022 (3)	0.033 (3)	0.026 (3)	−0.005 (2)	−0.002 (2)	0.004 (2)
C20	0.038 (3)	0.034 (3)	0.022 (3)	−0.003 (3)	−0.001 (3)	−0.002 (2)
C21	0.029 (3)	0.030 (3)	0.030 (3)	−0.001 (3)	−0.007 (2)	0.003 (3)
C22	0.038 (3)	0.025 (3)	0.027 (3)	−0.006 (3)	−0.003 (3)	0.007 (2)
C23	0.025 (3)	0.022 (3)	0.025 (3)	−0.001 (2)	−0.002 (2)	0.002 (2)
C24	0.025 (3)	0.026 (3)	0.025 (3)	0.000 (2)	0.001 (2)	0.001 (2)
C25	0.035 (3)	0.024 (3)	0.017 (3)	0.002 (3)	0.001 (2)	0.000 (2)
C26	0.027 (3)	0.031 (3)	0.018 (3)	0.006 (3)	0.001 (2)	−0.001 (2)
C27	0.024 (3)	0.036 (3)	0.015 (3)	−0.004 (3)	0.002 (2)	0.000 (2)
C28	0.026 (3)	0.039 (3)	0.023 (3)	−0.008 (3)	0.005 (2)	0.003 (3)
C29	0.025 (3)	0.033 (3)	0.025 (3)	−0.005 (3)	0.007 (2)	−0.001 (3)
C30	0.028 (3)	0.019 (3)	0.030 (3)	−0.004 (2)	−0.004 (2)	0.003 (2)
C31	0.034 (3)	0.036 (3)	0.025 (3)	−0.003 (3)	−0.005 (3)	−0.004 (3)
C32	0.025 (3)	0.029 (3)	0.028 (3)	0.004 (3)	0.000 (2)	0.002 (2)

### Geometric parameters (Å, º)

**Table:**

Os1—C25	1.929 (6)	N6—C12	1.481 (8)
Os1—C26	1.938 (6)	N7—C14	1.459 (10)
Os1—P1	2.2831 (13)	N7—C16	1.481 (9)
Os1—P2	2.2879 (14)	N7—C13	1.487 (8)
Os1—Os3	2.8861 (3)	N8—C17	1.464 (8)
Os1—Os2	2.8954 (3)	N8—C15	1.464 (9)
Os1A—C30	2.252 (12)	N8—C14	1.472 (9)
Os1A—C29	2.275 (12)	N9—C16	1.456 (8)
Os1A—P4	2.595 (10)	N9—C15	1.466 (9)
Os1A—P3	2.599 (10)	N9—C18	1.477 (8)
Os1A—Os3A	2.872 (15)	N10—C22	1.456 (8)
Os1A—Os2A	2.882 (15)	N10—C20	1.479 (8)
Os2—C28	1.880 (6)	N10—C19	1.481 (7)
Os2—C27	1.948 (6)	N11—C20	1.463 (8)
Os2—C29	1.957 (6)	N11—C21	1.473 (8)
Os2—P3	2.2965 (14)	N11—C23	1.473 (7)
Os2—Os3	2.9272 (3)	N12—C22	1.464 (8)
Os2A—C31	2.046 (13)	N12—C21	1.466 (8)
Os2A—C32	2.113 (12)	N12—C24	1.472 (7)
Os2A—C25	2.211 (12)	C1—H1A	0.9900
Os2A—P1	2.495 (11)	C1—H1B	0.9900
Os2A—Os3A	2.910 (15)	C2—H2A	0.9900
Os3—C31	1.880 (7)	C2—H2B	0.9900
Os3—C30	1.933 (6)	C3—H3A	0.9900
Os3—C32	1.951 (6)	C3—H3B	0.9900
Os3—P4	2.2963 (14)	C4—H4A	0.9900
Os3A—C28	1.866 (12)	C4—H4B	0.9900
Os3A—C27	2.092 (12)	C5—H5A	0.9900
Os3A—C26	2.193 (12)	C5—H5B	0.9900
Os3A—P2	2.425 (11)	C6—H6A	0.9900
P1—C6	1.848 (6)	C6—H6B	0.9900
P1—C5	1.850 (6)	C7—H7A	0.9900
P1—C1	1.864 (6)	C7—H7B	0.9900
P2—C7	1.841 (6)	C8—H8A	0.9900
P2—C11	1.854 (6)	C8—H8B	0.9900
P2—C12	1.861 (6)	C9—H9A	0.9900
P3—C18	1.830 (6)	C9—H9B	0.9900
P3—C13	1.849 (6)	C10—H10A	0.9900
P3—C17	1.851 (7)	C10—H10B	0.9900
P4—C24	1.841 (6)	C11—H11A	0.9900
P4—C19	1.849 (6)	C11—H11B	0.9900
P4—C23	1.852 (6)	C12—H12A	0.9900
O1—C25	1.153 (7)	C12—H12B	0.9900
O2—C26	1.147 (7)	C13—H13A	0.9900
O3—C27	1.144 (7)	C13—H13B	0.9900
O4—C28	1.154 (7)	C14—H14A	0.9900
O5—C29	1.130 (7)	C14—H14B	0.9900
O6—C30	1.146 (7)	C15—H15A	0.9900
O7—C31	1.146 (8)	C15—H15B	0.9900
O8—C32	1.146 (7)	C16—H16A	0.9900
N1—C2	1.469 (8)	C16—H16B	0.9900
N1—C4	1.472 (8)	C17—H17A	0.9900
N1—C1	1.477 (7)	C17—H17B	0.9900
N2—C2	1.459 (7)	C18—H18A	0.9900
N2—C3	1.468 (8)	C18—H18B	0.9900
N2—C5	1.476 (7)	C19—H19A	0.9900
N3—C4	1.457 (8)	C19—H19B	0.9900
N3—C3	1.465 (9)	C20—H20A	0.9900
N3—C6	1.473 (7)	C20—H20B	0.9900
N4—C8	1.452 (9)	C21—H21A	0.9900
N4—C7	1.462 (8)	C21—H21B	0.9900
N4—C10	1.470 (9)	C22—H22A	0.9900
N5—C8	1.470 (8)	C22—H22B	0.9900
N5—C9	1.471 (8)	C23—H23A	0.9900
N5—C11	1.476 (7)	C23—H23B	0.9900
N6—C9	1.448 (8)	C24—H24A	0.9900
N6—C10	1.465 (8)	C24—H24B	0.9900
			
C25—Os1—C26	176.7 (2)	C21—N12—C24	110.8 (5)
C25—Os1—P1	93.20 (17)	N1—C1—P1	112.7 (4)
C26—Os1—P1	88.80 (17)	N1—C1—H1A	109.1
C25—Os1—P2	90.29 (17)	P1—C1—H1A	109.1
C26—Os1—P2	91.96 (17)	N1—C1—H1B	109.1
P1—Os1—P2	100.38 (5)	P1—C1—H1B	109.1
C25—Os1—Os3	79.18 (16)	H1A—C1—H1B	107.8
C26—Os1—Os3	97.76 (16)	N2—C2—N1	114.4 (5)
P1—Os1—Os3	103.89 (4)	N2—C2—H2A	108.7
P2—Os1—Os3	153.97 (4)	N1—C2—H2A	108.7
C25—Os1—Os2	102.28 (16)	N2—C2—H2B	108.7
C26—Os1—Os2	74.96 (16)	N1—C2—H2B	108.7
P1—Os1—Os2	154.98 (4)	H2A—C2—H2B	107.6
P2—Os1—Os2	99.09 (4)	N3—C3—N2	114.5 (5)
Os3—Os1—Os2	60.835 (8)	N3—C3—H3A	108.6
C30—Os1A—C29	158.1 (6)	N2—C3—H3A	108.6
C30—Os1A—P4	76.0 (3)	N3—C3—H3B	108.6
C29—Os1A—P4	112.6 (4)	N2—C3—H3B	108.6
C30—Os1A—P3	115.9 (4)	H3A—C3—H3B	107.6
C29—Os1A—P3	76.6 (3)	N3—C4—N1	114.5 (5)
P4—Os1A—P3	124.8 (4)	N3—C4—H4A	108.6
C30—Os1A—Os3A	95.2 (4)	N1—C4—H4A	108.6
C29—Os1A—Os3A	66.8 (4)	N3—C4—H4B	108.6
P4—Os1A—Os3A	148.9 (5)	N1—C4—H4B	108.6
P3—Os1A—Os3A	86.0 (4)	H4A—C4—H4B	107.6
C30—Os1A—Os2A	66.8 (4)	N2—C5—P1	113.5 (4)
C29—Os1A—Os2A	92.8 (4)	N2—C5—H5A	108.9
P4—Os1A—Os2A	88.7 (4)	P1—C5—H5A	108.9
P3—Os1A—Os2A	146.5 (5)	N2—C5—H5B	108.9
Os3A—Os1A—Os2A	60.8 (4)	P1—C5—H5B	108.9
C28—Os2—C27	95.9 (3)	H5A—C5—H5B	107.7
C28—Os2—C29	93.3 (3)	N3—C6—P1	113.6 (4)
C27—Os2—C29	170.4 (3)	N3—C6—H6A	108.9
C28—Os2—P3	95.26 (18)	P1—C6—H6A	108.9
C27—Os2—P3	91.00 (16)	N3—C6—H6B	108.9
C29—Os2—P3	90.61 (17)	P1—C6—H6B	108.9
C28—Os2—Os1	95.78 (18)	H6A—C6—H6B	107.7
C27—Os2—Os1	77.69 (16)	N4—C7—P2	113.6 (4)
C29—Os2—Os1	98.95 (17)	N4—C7—H7A	108.8
P3—Os2—Os1	164.93 (4)	P2—C7—H7A	108.8
C28—Os2—Os3	153.25 (19)	N4—C7—H7B	108.8
C27—Os2—Os3	88.68 (18)	P2—C7—H7B	108.8
C29—Os2—Os3	81.95 (17)	H7A—C7—H7B	107.7
P3—Os2—Os3	111.03 (4)	N4—C8—N5	115.6 (5)
Os1—Os2—Os3	59.425 (8)	N4—C8—H8A	108.4
C31—Os2A—C32	85.1 (5)	N5—C8—H8A	108.4
C31—Os2A—C25	103.2 (5)	N4—C8—H8B	108.4
C32—Os2A—C25	163.9 (6)	N5—C8—H8B	108.4
C31—Os2A—P1	115.8 (5)	H8A—C8—H8B	107.4
C32—Os2A—P1	108.0 (5)	N6—C9—N5	114.9 (5)
C25—Os2A—P1	81.2 (4)	N6—C9—H9A	108.5
C31—Os2A—Os1A	91.8 (5)	N5—C9—H9A	108.5
C32—Os2A—Os1A	65.8 (4)	N6—C9—H9B	108.5
C25—Os2A—Os1A	99.7 (5)	N5—C9—H9B	108.5
P1—Os2A—Os1A	151.6 (5)	H9A—C9—H9B	107.5
C31—Os2A—Os3A	147.1 (6)	N6—C10—N4	114.3 (5)
C32—Os2A—Os3A	95.8 (5)	N6—C10—H10A	108.7
C25—Os2A—Os3A	69.7 (4)	N4—C10—H10A	108.7
P1—Os2A—Os3A	95.3 (4)	N6—C10—H10B	108.7
Os1A—Os2A—Os3A	59.4 (4)	N4—C10—H10B	108.7
C31—Os3—C30	92.2 (3)	H10A—C10—H10B	107.6
C31—Os3—C32	94.4 (3)	N5—C11—P2	113.9 (4)
C30—Os3—C32	173.0 (2)	N5—C11—H11A	108.8
C31—Os3—P4	94.47 (19)	P2—C11—H11A	108.8
C30—Os3—P4	89.81 (17)	N5—C11—H11B	108.8
C32—Os3—P4	92.11 (18)	P2—C11—H11B	108.8
C31—Os3—Os1	97.47 (19)	H11A—C11—H11B	107.7
C30—Os3—Os1	97.74 (17)	N6—C12—P2	113.0 (4)
C32—Os3—Os1	79.00 (17)	N6—C12—H12A	109.0
P4—Os3—Os1	165.60 (4)	P2—C12—H12A	109.0
C31—Os3—Os2	155.42 (19)	N6—C12—H12B	109.0
C30—Os3—Os2	82.79 (18)	P2—C12—H12B	109.0
C32—Os3—Os2	90.20 (17)	H12A—C12—H12B	107.8
P4—Os3—Os2	109.50 (4)	N7—C13—P3	111.5 (4)
Os1—Os3—Os2	59.740 (8)	N7—C13—H13A	109.3
C28—Os3A—C27	91.7 (5)	P3—C13—H13A	109.3
C28—Os3A—C26	104.2 (5)	N7—C13—H13B	109.3
C27—Os3A—C26	155.8 (6)	P3—C13—H13B	109.3
C28—Os3A—P2	114.0 (5)	H13A—C13—H13B	108.0
C27—Os3A—P2	107.9 (5)	N7—C14—N8	115.7 (6)
C26—Os3A—P2	82.5 (4)	N7—C14—H14A	108.3
C28—Os3A—Os1A	95.4 (5)	N8—C14—H14A	108.3
C27—Os3A—Os1A	63.4 (4)	N7—C14—H14B	108.3
C26—Os3A—Os1A	96.5 (4)	N8—C14—H14B	108.3
P2—Os3A—Os1A	150.0 (5)	H14A—C14—H14B	107.4
C28—Os3A—Os2A	149.2 (6)	N8—C15—N9	114.9 (5)
C27—Os3A—Os2A	92.4 (4)	N8—C15—H15A	108.6
C26—Os3A—Os2A	64.6 (4)	N9—C15—H15A	108.6
P2—Os3A—Os2A	93.7 (4)	N8—C15—H15B	108.6
Os1A—Os3A—Os2A	59.8 (4)	N9—C15—H15B	108.6
C6—P1—C5	97.7 (3)	H15A—C15—H15B	107.5
C6—P1—C1	96.5 (3)	N9—C16—N7	114.3 (5)
C5—P1—C1	96.4 (3)	N9—C16—H16A	108.7
C6—P1—Os1	115.11 (19)	N7—C16—H16A	108.7
C5—P1—Os1	122.0 (2)	N9—C16—H16B	108.7
C1—P1—Os1	123.49 (18)	N7—C16—H16B	108.7
C6—P1—Os2A	134.6 (3)	H16A—C16—H16B	107.6
C5—P1—Os2A	127.4 (3)	N8—C17—P3	112.8 (5)
C1—P1—Os2A	84.2 (3)	N8—C17—H17A	109.0
C7—P2—C11	96.8 (3)	P3—C17—H17A	109.0
C7—P2—C12	97.7 (3)	N8—C17—H17B	109.0
C11—P2—C12	95.7 (3)	P3—C17—H17B	109.0
C7—P2—Os1	119.0 (2)	H17A—C17—H17B	107.8
C11—P2—Os1	120.71 (19)	N9—C18—P3	113.3 (4)
C12—P2—Os1	121.3 (2)	N9—C18—H18A	108.9
C7—P2—Os3A	103.5 (3)	P3—C18—H18A	108.9
C11—P2—Os3A	159.4 (3)	N9—C18—H18B	108.9
C12—P2—Os3A	85.5 (3)	P3—C18—H18B	108.9
C18—P3—C13	98.2 (3)	H18A—C18—H18B	107.7
C18—P3—C17	97.6 (3)	N10—C19—P4	112.1 (4)
C13—P3—C17	97.9 (3)	N10—C19—H19A	109.2
C18—P3—Os2	121.42 (19)	P4—C19—H19A	109.2
C13—P3—Os2	120.9 (2)	N10—C19—H19B	109.2
C17—P3—Os2	115.8 (2)	P4—C19—H19B	109.2
C18—P3—Os1A	87.4 (3)	H19A—C19—H19B	107.9
C13—P3—Os1A	111.8 (3)	N11—C20—N10	114.5 (5)
C17—P3—Os1A	148.9 (3)	N11—C20—H20A	108.6
C24—P4—C19	97.8 (3)	N10—C20—H20A	108.6
C24—P4—C23	96.9 (3)	N11—C20—H20B	108.6
C19—P4—C23	98.3 (3)	N10—C20—H20B	108.6
C24—P4—Os3	114.33 (19)	H20A—C20—H20B	107.6
C19—P4—Os3	122.26 (19)	N12—C21—N11	114.5 (5)
C23—P4—Os3	121.95 (19)	N12—C21—H21A	108.6
C24—P4—Os1A	151.2 (3)	N11—C21—H21A	108.6
C19—P4—Os1A	90.1 (3)	N12—C21—H21B	108.6
C23—P4—Os1A	109.3 (3)	N11—C21—H21B	108.6
C2—N1—C4	108.3 (5)	H21A—C21—H21B	107.6
C2—N1—C1	111.4 (5)	N10—C22—N12	115.0 (5)
C4—N1—C1	110.9 (5)	N10—C22—H22A	108.5
C2—N2—C3	108.5 (5)	N12—C22—H22A	108.5
C2—N2—C5	110.9 (5)	N10—C22—H22B	108.5
C3—N2—C5	110.8 (5)	N12—C22—H22B	108.5
C4—N3—C3	108.9 (5)	H22A—C22—H22B	107.5
C4—N3—C6	110.0 (5)	N11—C23—P4	112.6 (4)
C3—N3—C6	111.3 (5)	N11—C23—H23A	109.1
C8—N4—C7	111.6 (5)	P4—C23—H23A	109.1
C8—N4—C10	107.9 (5)	N11—C23—H23B	109.1
C7—N4—C10	110.7 (5)	P4—C23—H23B	109.1
C8—N5—C9	107.0 (5)	H23A—C23—H23B	107.8
C8—N5—C11	110.2 (5)	N12—C24—P4	113.1 (4)
C9—N5—C11	111.2 (5)	N12—C24—H24A	109.0
C9—N6—C10	109.1 (5)	P4—C24—H24A	109.0
C9—N6—C12	110.5 (5)	N12—C24—H24B	109.0
C10—N6—C12	111.0 (5)	P4—C24—H24B	109.0
C14—N7—C16	108.1 (6)	H24A—C24—H24B	107.8
C14—N7—C13	110.6 (6)	O1—C25—Os1	175.5 (5)
C16—N7—C13	111.3 (5)	O1—C25—Os2A	135.8 (6)
C17—N8—C15	111.0 (5)	O2—C26—Os1	175.0 (5)
C17—N8—C14	110.6 (6)	O2—C26—Os3A	130.9 (5)
C15—N8—C14	107.6 (6)	O3—C27—Os2	175.6 (5)
C16—N9—C15	109.4 (5)	O3—C27—Os3A	137.6 (5)
C16—N9—C18	110.0 (5)	O4—C28—Os3A	129.4 (6)
C15—N9—C18	110.6 (5)	O4—C28—Os2	176.3 (6)
C22—N10—C20	109.0 (5)	O5—C29—Os2	175.0 (5)
C22—N10—C19	110.6 (5)	O5—C29—Os1A	134.3 (6)
C20—N10—C19	110.7 (5)	O6—C30—Os3	173.8 (5)
C20—N11—C21	108.2 (5)	O6—C30—Os1A	134.3 (6)
C20—N11—C23	110.9 (5)	O7—C31—Os3	178.1 (6)
C21—N11—C23	111.1 (5)	O7—C31—Os2A	132.5 (6)
C22—N12—C21	108.3 (5)	O8—C32—Os3	176.2 (5)
C22—N12—C24	110.9 (5)	O8—C32—Os2A	136.4 (6)
			
C2—N1—C1—P1	60.5 (5)	C17—P3—C13—N7	−50.2 (5)
C4—N1—C1—P1	−60.1 (6)	Os2—P3—C13—N7	−176.8 (4)
C6—P1—C1—N1	49.1 (5)	Os1A—P3—C13—N7	139.2 (5)
C5—P1—C1—N1	−49.4 (5)	C16—N7—C14—N8	54.6 (7)
Os1—P1—C1—N1	175.1 (3)	C13—N7—C14—N8	−67.5 (8)
Os2A—P1—C1—N1	−176.6 (5)	C17—N8—C14—N7	66.7 (8)
C3—N2—C2—N1	−55.2 (6)	C15—N8—C14—N7	−54.7 (7)
C5—N2—C2—N1	66.7 (7)	C17—N8—C15—N9	−67.3 (7)
C4—N1—C2—N2	55.2 (6)	C14—N8—C15—N9	53.9 (7)
C1—N1—C2—N2	−67.0 (6)	C16—N9—C15—N8	−54.7 (7)
C4—N3—C3—N2	−54.1 (6)	C18—N9—C15—N8	66.7 (7)
C6—N3—C3—N2	67.4 (6)	C15—N9—C16—N7	54.0 (7)
C2—N2—C3—N3	54.5 (6)	C18—N9—C16—N7	−67.8 (7)
C5—N2—C3—N3	−67.5 (6)	C14—N7—C16—N9	−53.6 (7)
C3—N3—C4—N1	54.2 (6)	C13—N7—C16—N9	68.1 (8)
C6—N3—C4—N1	−68.0 (6)	C15—N8—C17—P3	60.0 (7)
C2—N1—C4—N3	−54.6 (6)	C14—N8—C17—P3	−59.4 (7)
C1—N1—C4—N3	67.9 (6)	C18—P3—C17—N8	−49.2 (5)
C2—N2—C5—P1	−61.1 (6)	C13—P3—C17—N8	50.3 (5)
C3—N2—C5—P1	59.5 (6)	Os2—P3—C17—N8	−179.6 (4)
C6—P1—C5—N2	−47.5 (5)	Os1A—P3—C17—N8	−146.8 (6)
C1—P1—C5—N2	49.9 (5)	C16—N9—C18—P3	61.2 (6)
Os1—P1—C5—N2	−173.6 (3)	C15—N9—C18—P3	−59.8 (6)
Os2A—P1—C5—N2	137.6 (5)	C13—P3—C18—N9	−50.1 (5)
C4—N3—C6—P1	61.8 (6)	C17—P3—C18—N9	49.1 (5)
C3—N3—C6—P1	−59.1 (6)	Os2—P3—C18—N9	175.7 (3)
C5—P1—C6—N3	47.2 (5)	Os1A—P3—C18—N9	−161.7 (5)
C1—P1—C6—N3	−50.2 (5)	C22—N10—C19—P4	−60.8 (6)
Os1—P1—C6—N3	178.0 (4)	C20—N10—C19—P4	60.1 (6)
Os2A—P1—C6—N3	−138.5 (5)	C24—P4—C19—N10	49.3 (5)
C8—N4—C7—P2	59.8 (6)	C23—P4—C19—N10	−48.9 (5)
C10—N4—C7—P2	−60.5 (6)	Os3—P4—C19—N10	174.7 (3)
C11—P2—C7—N4	−48.7 (5)	Os1A—P4—C19—N10	−158.4 (5)
C12—P2—C7—N4	48.1 (5)	C21—N11—C20—N10	−54.0 (6)
Os1—P2—C7—N4	−179.5 (4)	C23—N11—C20—N10	68.0 (7)
Os3A—P2—C7—N4	135.3 (5)	C22—N10—C20—N11	53.6 (6)
C7—N4—C8—N5	−66.4 (7)	C19—N10—C20—N11	−68.3 (7)
C10—N4—C8—N5	55.5 (7)	C22—N12—C21—N11	−55.3 (6)
C9—N5—C8—N4	−55.4 (7)	C24—N12—C21—N11	66.5 (6)
C11—N5—C8—N4	65.6 (7)	C20—N11—C21—N12	55.4 (6)
C10—N6—C9—N5	−55.0 (6)	C23—N11—C21—N12	−66.6 (6)
C12—N6—C9—N5	67.3 (6)	C20—N10—C22—N12	−53.8 (6)
C8—N5—C9—N6	54.6 (6)	C19—N10—C22—N12	68.1 (6)
C11—N5—C9—N6	−65.7 (6)	C21—N12—C22—N10	54.6 (6)
C9—N6—C10—N4	54.3 (7)	C24—N12—C22—N10	−67.1 (6)
C12—N6—C10—N4	−67.7 (7)	C20—N11—C23—P4	−59.9 (6)
C8—N4—C10—N6	−54.0 (7)	C21—N11—C23—P4	60.4 (5)
C7—N4—C10—N6	68.4 (7)	C24—P4—C23—N11	−50.1 (4)
C8—N5—C11—P2	−59.1 (6)	C19—P4—C23—N11	48.8 (5)
C9—N5—C11—P2	59.4 (6)	Os3—P4—C23—N11	−174.6 (3)
C7—P2—C11—N5	49.0 (5)	Os1A—P4—C23—N11	141.9 (4)
C12—P2—C11—N5	−49.5 (5)	C22—N12—C24—P4	59.2 (6)
Os1—P2—C11—N5	178.6 (3)	C21—N12—C24—P4	−61.1 (6)
Os3A—P2—C11—N5	−142.1 (8)	C19—P4—C24—N12	−48.9 (4)
C9—N6—C12—P2	−62.4 (6)	C23—P4—C24—N12	50.6 (4)
C10—N6—C12—P2	58.8 (6)	Os3—P4—C24—N12	−179.6 (3)
C7—P2—C12—N6	−47.0 (5)	Os1A—P4—C24—N12	−153.5 (5)
C11—P2—C12—N6	50.7 (5)	C27—Os3A—C28—O4	125.6 (8)
Os1—P2—C12—N6	−177.9 (3)	C26—Os3A—C28—O4	−72.8 (9)
Os3A—P2—C12—N6	−150.0 (5)	P2—Os3A—C28—O4	15.1 (10)
C14—N7—C13—P3	60.7 (6)	Os1A—Os3A—C28—O4	−171.0 (7)
C16—N7—C13—P3	−59.5 (7)	Os2A—Os3A—C28—O4	−136.8 (10)
C18—P3—C13—N7	48.7 (5)		
